# Hydroethanolic Extract of *Morus nigra* L. Leaves: A Dual PPAR-α/γ Agonist with Anti-Inflammatory Properties in Lipopolysaccharide-Stimulated RAW 264.7

**DOI:** 10.3390/plants11223147

**Published:** 2022-11-17

**Authors:** Amanda de Assis Carneiro, Simone Batista Pires Sinoti, Marcela Medeiros de Freitas, Luiz Alberto Simeoni, Christopher William Fagg, Pérola de Oliveira Magalhães, Dâmaris Silveira, Yris Maria Fonseca-Bazzo

**Affiliations:** 1 Natural Products Laboratory, Department of Pharmacy, Health Sciences School, University of Brasília (UnB), Campus Darcy Ribeiro, Asa Norte, Brasilia 70910-900, Brazil; 2 Molecular Pharmacology Laboratory, Department of Pharmacy, Health Sciences School, University of Brasília (UnB), Campus Darcy Ribeiro, Asa Norte, Brasilia 70910-900, Brazil; 3 Department of Botany, Institute of Biological Science, School of Pharmacy, Ceilândia Campus, University of Brasília, Brasilia 70910-900, Brazil

**Keywords:** Herbal medicines, *Morus nigra*, anti-inflammatory activity, PPAR

## Abstract

Inhibition of systemic inflammation has been a beneficial strategy in treating several non-communicable diseases, which represent one of the major causes of mortality in the world. The Peroxisome Proliferator-Activated Receptors (PPAR) are interesting pharmacological targets, since they can act both through the metabolic and anti-inflammatory pathways. *Morus nigra* L. has flavonoids in its chemical composition with recognized anti-oxidant activity and often associated with anti-inflammatory activity. Therefore, this study aimed to evaluate the hydroethanolic extract of *M. nigra* leaves’ ability to activate PPAR and promote anti-inflammatory effects in lipopolysaccharide (LPS)-stimulated murine macrophage cells. The leaf extract was prepared by cold maceration, and the chemical profile was obtained by HPLC-DAD. Activation of PPAR α and γ was evaluated by the luciferase reporter assay. The anti-inflammatory activity was assessed by measuring the reactive oxygen species (ROS), nitric oxide (NO), and Tumor Necrosis Factor-α (TNF-α) in RAW 264.7 cells after stimulation with LPS from *Escherichia coli*. The HPLC-DAD analysis identified two major compounds: rutin and isoquercitrin. The extract showed agonist activity for the two types of PPAR, α and γ, although its major compounds, rutin and isoquercitrin, did not significantly activate the receptors. In addition, the extract significantly reduced the production of ROS, NO, and TNF-α. Treatment with the specific PPAR-α antagonist, GW 6471, was able to partially block the anti-inflammatory effect caused by the extract.

## 1. Introduction

Exposure to harmful stimuli, such as pathogens, toxins, and cell damage, triggers a series of complex events that are included in the inflammatory response. This response is essential as a natural protective and restorative measure; however, the prolongation of inflammation and failure to resolve it can cause damage and contribute to the maintenance of chronic diseases [1,2].

Besides the chronic inflammation that develops after an unresolved acute phase, triggered by classic noxious stimuli, there is also chronic systemic inflammation that, in most cases, is related to changes in homeostatic conditions. This general inflammatory state is associated with several non-communicable diseases (NCD), such as atherosclerosis, type II diabetes, neurodegenerative diseases, and cancer [3,4], which have a significant impact on public health since they represent one of the major causes of mortality in the world [5,6].

The NCD often present an increase in oxidative stress, pro-inflammatory cytokines, and acute phase proteins [3,4,5]. Furthermore, the inhibition of inflammation has been shown to be beneficial in managing these diseases [7,8].

Peroxisome proliferator-activated receptors (PPAR) are nuclear receptors that function as transcription factors regulating the expression of several genes. These receptors act mainly on lipid and glucose metabolism. However, in recent years, PPAR, mainly the α and γ subtypes, have been associated with anti-inflammatory activity due to their ability to inhibit the expression of inflammatory cytokines and promote immune cell differentiation. Thus, these receptors constitute relevant pharmacological targets for new anti-inflammatory drugs, especially in the case of chronic diseases related to a general chronic inflammatory state, since PPAR can act both through the metabolic and anti-inflammatory pathways [9,10,11].

Traditional medicine is a health resource based on the experience and long-term practices of different cultures. The World Health Organization (WHO) recommends the inclusion of traditional and complementary medicine in health systems and believes that carrying out these practices with safety and quality can meet the communities need for sustainable and culturally sensitive primary care. In addition, it is especially important as a tool for preventing and managing lifestyle-related chronic diseases [12,13].

Medicinal plants and herbal medicines are one of the most used traditional and complementary medicine resources in the world. Increased demand for these products includes accessibility, cultural influence, a greater sense of security, and increased scientific knowledge [13,14].

The leaves of *Morus nigra* L., popularly known as mulberry, are traditionally used as a tea, mainly for the relief of climacteric symptoms [15], cholesterol reduction, weight loss [16,17], and diabetes control [18,19]. In its composition, the presence of rutin, quercetin, kaempferol, quercitrin, and chlorogenic acid has already been reported [20,21,22]. These substances have recognized anti-oxidant activity and are often related to anti-inflammatory activity [23,24]. Therefore, this study aimed to evaluate the ability of the hydroethanolic extract of *Morus nigra* leaves (HEMNL) to activate PPAR and promote anti-inflammatory effects in lipopolysaccharide (LPS)-stimulated murine macrophage cells.

## 2. Results

### 2.1. Chemical Profile

**Figure 1:**
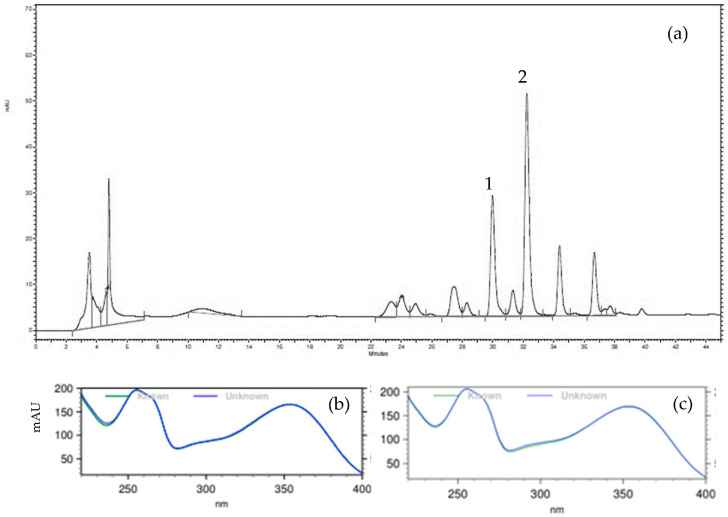
(**a**) Chromatogram of hydroethanolic extract of *Morus nigra* leaves, obtained by high performance liquid chromatography with a diode array detector (HPLC-DAD). Analysis performed by gradient elution with acetonitrile and phosphoric acid and detection at 354 nm. (**b**): spectrum of the peak 1 (“un-known” line) referring to the rutin superimposed on the spectrum of the standard (“known” line); (**c**): spectrum of the peak 2 (“unknown” line) referring to isoquercitrin superimposed on the spectrum of the standard (“known” line).

The chemical profile of HEMNL was obtained by High Performance Liquid Chromatography with Diode Array Detector (HPLC-DAD) according to the method previously validated for the standardized hydroethanolic extract of *M. nigra* leaves [20]. The compounds rutin, isoquercitrin, and chlorogenic acid were previously chosen as markers for the standardized extract, also according to the previous study [20]. The chemical profile (Figure 1a) was similar to the profile found in work by [20], with four major peaks, of which two were identified: rutin (3.74 µg/mg, Figure 1b and isoquercitrin (5.92 µg/mg, Figure 1c). However, chlorogenic acid, one of the phytochemicals markers of the standardized extract described previously by [20], was not detected.

### 2.2. Extract Cytotoxicity

The extract cytotoxicity was evaluated by the 3-[4,5-dimethylthiazol-2-yl]-2,5 diphenyl tetrazolium bromide (MTT) reduction method. Extract concentrations were considered cytotoxic when a viability reduction was greater than 30%. Therefore, only concentrations that presented a viability greater than 70% were used for future tests, as recommended by ISO 10993-5 [25].

**Figure 2:**
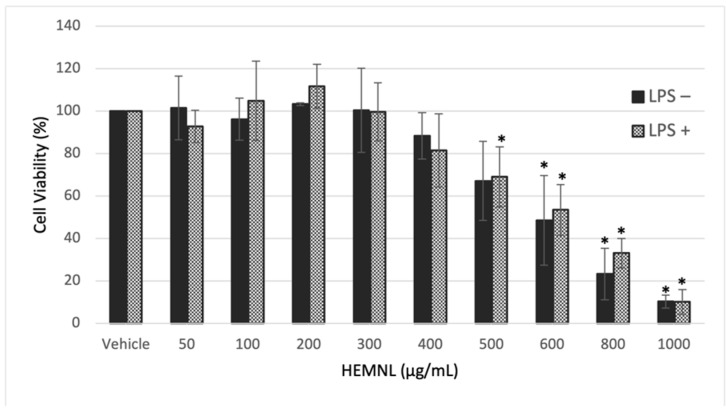
Cytotoxicity of hydroethanolic extract of *Morus nigra* leaves (HEMN) in RAW 264.7 cells with (LPS+) or without (LPS−) lipopolysaccharide-stimulation. Vehicle: DMEM with 2% of ethanol. Data represent the mean of three experiments performed in triplicate ± standard deviation. Statistical analysis was performed by one-way ANOVA and Tukey’s post-test. * *p* < 0.005 in relation to vehicle.

**Figure 3:**
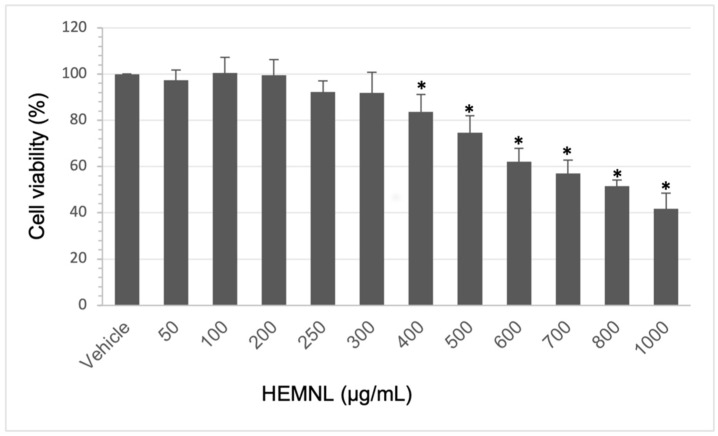
Cytotoxicity of hydroethanolic extract of *Morus nigra* leaves in HeLa cells. Data represent the mean of three experiments performed in triplicate ± standard deviation. Vehicle: DMEM with 2% of ethanol. Statistical analysis was performed by one-way ANOVA test followed by Tukey’s post-test. * *p* < 0.005 in relation to the vehicle.

The HEMNL showed cytotoxicity in the tested cell lines only at concentrations greater than 500 µg/mL. The concentration needed to inhibit 50% of cell viability (IC_50_) values found were 597.1 ± 103.70 µg/mL for RAW 264.7 without LPS stimulation, 622.2 ± 79.04 µg/mL for RAW264.7 stimulated with LPS (Figure 2), and 562.23 ± 32.24 µg/mL for HeLa (Figure 3). Even with the macrophage cells undergoing an inflammatory process caused by LPS, the extract maintained the cytotoxicity profile similar to non-stimulated cells, presenting an IC_50_ value with no statistical difference (Mann–Whitney test) to the non-stimulated cells (*p* > 0.005).

### 2.3. Activation of Nuclear Receptors PPAR-γ and PPAR-α

**Figure 4:**
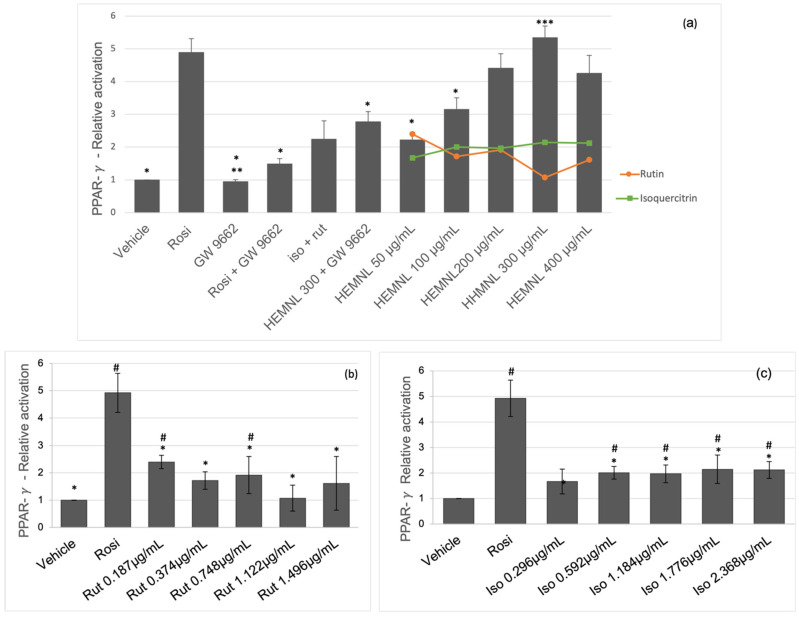
Activation of PPAR-γ evaluated by the Luciferase Reporter Assay. Rosi: Rosiglitazone 10^−5^ M—PPAR-γ agonist; GW 9662 10^−5^ M—PPAR-γ antagonist. Iso + Rut: Isoquercitrin 1.77 µg/mL and rutin 1.12 µg/mL; HEMNL: hydroethanolic extract of *Morus nigra* leaves; Vehicle: DMEM with 2% of ethanol. (**a**) Activation of PPAR-γ by HEMNL compared to the activation by rutin (orange line) or isoquercitrin (green line) tested separately at concentrations corresponding to their presence in the extract. (**b**) Activation of PPAR-γ by rutin. (**c**) Activation of PPAR-γ by isoquercitrin. Data represent mean ± standard deviation. Statistical analysis performed by one-way ANOVA test followed by Tukey’s post-test. * *p* < 0.001 in relation to Rosi. ** *p* < 0.001 in relation to Rosi, HEMNL 300 + GW 9662, HEMNL 50, 100, 200, 300, and 400 µg/mL and iso + rut. *** *p* < 0.001 in relation to HEMNL 300 + GW 9662, iso + rut, vehicle, GW 9662, HEMNL 50 and 100 µg/mL. # *p* < 0.05 in relation to vehicle.

The nuclear receptor activation was evaluated by the Luciferase reporter assay. The activation of PPAR-γ by the extract was compared to a full agonist of PPAR-γ control, rosiglitazone, an anti-diabetic drug. The HEMNL at concentrations of 200, 300, and 400 μg/mL was as effective as rosiglitazone in activating the PPAR-γ receptor (*p* > 0.005) (Figure 4a). The cells treated with the extract at the concentration of 400 μg/mL showed cell death, above the expected, observed under the microscope. Which may have been caused by the greater sensitivity of the cells when they are transfected. This may explain the reduction in receptor activation for this extract concentration.

Despite the activation of PPAR-γ after simultaneous treatment with the HEMNL (300 μg/mL) and the substance GW 9662 (selective irreversible PPAR-γ antagonist), there was a significant reduction (*p* < 0.001) of 48% in relation to the treatment with the extract-only at the same concentration (HEMNL 300 μg/mL) (Figure 4a).

The substances isoquercitrin and rutin were tested separately, at concentrations corresponding to their presence in the extract, to evaluate their roles in activating the PPAR-γ receptor. Rutin could activate the receptor only at concentrations of 0.187 and 0.748 μg/mL (*p* < 0.05), corresponding to 50 and 200 μg/mL of the extract, respectively. However, the activation rate remained low even with increasing concentration. In addition, the dose of 0.748 μg/mL led to an activation rate 56% lower than the corresponding concentration of the extract (Figure 4b).

Despite activating the receptor PPAR-γ, the activation rate caused by isoquercitrin remained linear, even with the increase in the concentration. In addition, it was significantly lower than the activation rate caused by the extract (*p* < 0.0001 in the one-way ANOVA test followed by the Tukey post-test) (Figure 4c). Additionally, rutin and isoquercitrin were tested together at concentrations corresponding to 300 μg/mL of the extract; however, the activation rate was 58% lower than that caused by the crude extract (*p* < 0.0001) (Figure 4a).

**Figure 5:**
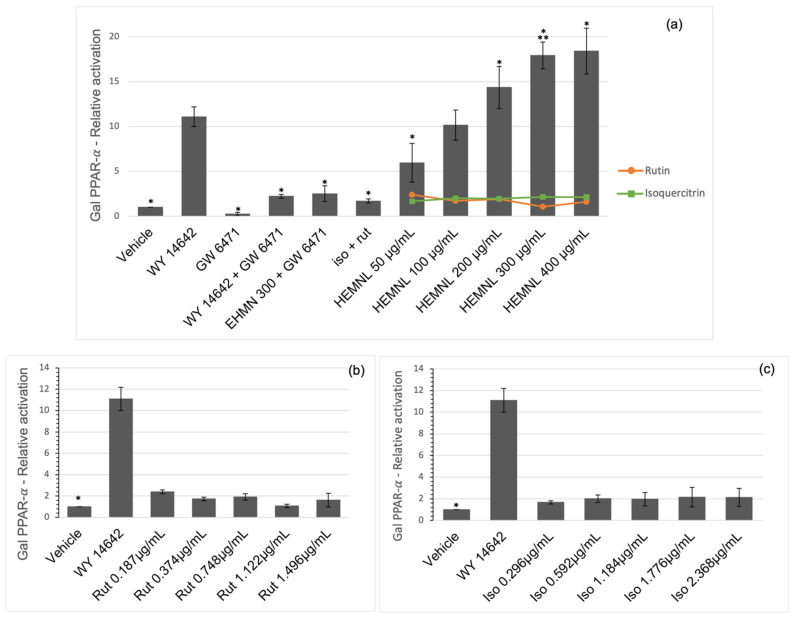
Activation of PPAR-α evaluated by the Luciferase Reporter Assay. WY 14642: 10^−5^ M—PPAR-α agonist; GW 6471 10^−4^ M—PPAR-α antagonist. Iso + Rut: Isoquercitrin 1.77 µg/mL and rutin 1.12 µg/mL; HEMNL: Hydroethanolic extract of *Morus nigra* leaves; Vehicle: DMEM with 2% of ethanol. (**a**): Activation of PPAR-α by HEMNL compared to activating by rutin (orange line) or isoquercitrin (green line) tested separately at concentrations corresponding to their presence in the extract. (**b**) Activation of PPAR-α by rutin. (**c**) Activation of PPAR-α by isoquercitrin. Data represent mean ± standard deviation. Statistical analysis was performed by one-way ANOVA and Tukey’s post-test. * *p* < 0.0001 in relation to WY 14642. ** *p* < 0.0001 in relation to WY 14642, iso + rut and HEMNL 300 + GW 6471.

Activation of PPAR-α by the extract was compared with the positive control, WY 14642, full agonist of PPAR-α. The extract was able to activate the receptor in a dose-dependent manner. Furthermore, at a concentration of 100 μg/mL, the extract was as effective as WY 14642 (*p* = 0.9739), and at concentrations of 200, 300, and 400 μg/mL, a higher activation rate than WY 14642 (*p* < 0.002) was reached (Figure 5a). However, the substances isoquercitrin and rutin, tested alone or together, did not activate the PPAR-α receptor (*p* > 0.05) (Figure 5b,c).

While the simultaneous treatment with the extract (300 μg/mL) and the PPAR-α antagonist, GW 6471, reduced the effect of the extract by 86%, showing no statistical difference (*p* > 0.99) in relation to the simultaneous treatment of the agonist (WY 14642) and antagonist (GW 6471) (Figure 5a).

### 2.4. Intracellular ROS Reduction

**Figure 6:**
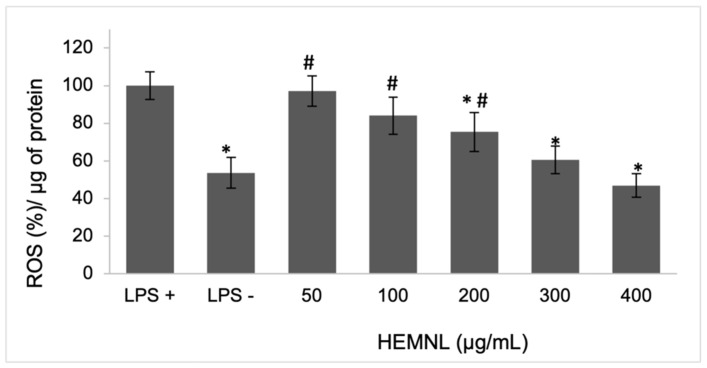
Inhibition of the production of reactive oxygen species in lipopolysaccharide (LPS)-stimulated cells. LPS+: positive control—cells stimulated with LPS and treated with vehicle (DMEM with 2% of ethanol). LPS−: negative control—cells not stimulated with LPS and treated with vehicle. Data represent mean ± standard deviation. * *p* < 0.001 in relation to LPS+; # *p* < 0.001 in relation to LPS−.

The assessment of intracellular production of ROS was performed using the 2,7-dichlorofluorescein diacetate (DCFH-DA) reagent method. The extract of *M. nigra* leaves at 200, 300, and 400 µg/mL reduced ROS production by 25, 39, and 53%, respectively, in LPS-stimulated cells compared to untreated cells. The 50 and 100 µg/mL doses, although showing a reduction (3 and 16%, respectively), were not significant when analyzed by the one-way ANOVA statistical test followed by the Tukey post-test (Figure 6).

### 2.5. Reduction of NO Production

**Figure 7:**
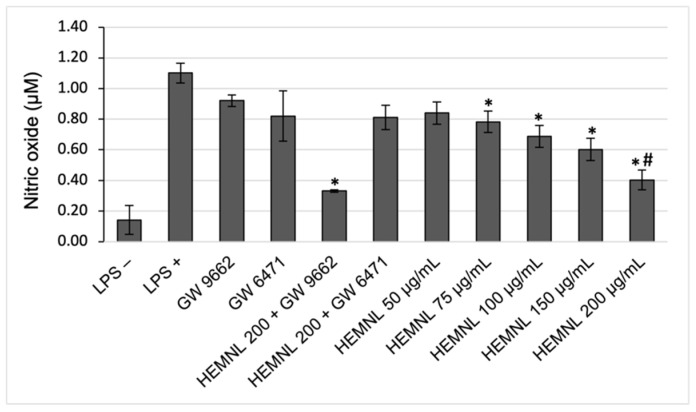
Inhibition of nitric oxide production in lipopolysaccharide (LPS)-stimulated RAW 264.7 cells, evaluated by the Griess method. LPS+: positive control—cells stimulated with LPS and treated with vehicle (DMEM with 2% of ethanol). LPS−: negative control—cells not stimulated with LPS and treated with vehicle. HEMNL: hydroethanolic extract of *Morus nigra* leaves. Data represent mean ± standard deviation. Statistical analysis was performed by one-way ANOVA and Tukey’s post-test. * *p* < 0.039 in relation to LPS+; # *p* = 0.002 in relation to HEMNL 200 + GW 6471.

The stimulation with LPS was effective in increasing the production of nitric oxide in RAW 264.7 cells. After treatment with the extract, at concentrations from 75 to 200 μg/mL, NO production was significantly reduced (*p* < 0.04 concerning LPS-stimulated and untreated cells) in 28.86 to 63.40%. Moreover, there was no significant NO production reduction in LPS-stimulated cells (*p* = 0.0863 in relation to LPS-stimulated and untreated cells) under concomitant treatment with the extract (200 μg/mL) and the PPAR-α antagonist, GW 6471. Therefore, the simultaneous treatment with the extract (200 μg/mL) and the PPAR-γ antagonist, GW 9662, showed no significant difference in relation to treatment with the extract only (*p* = 0.9995) (Figure 7).

### 2.6. Reduction of Pro-Inflammatory Cytokine TNF-α

**Figure 8:**
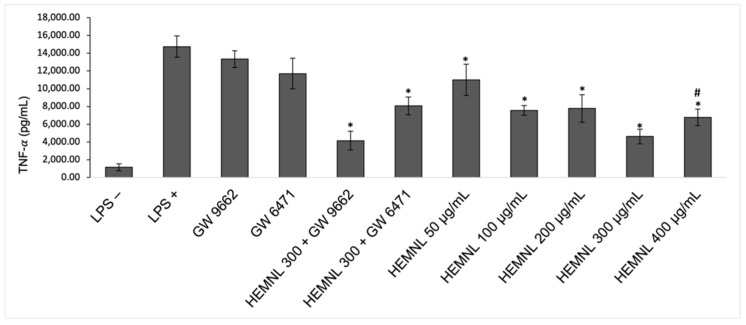
Reduction of tumor necrosis factor α (TNF-α) production in lipopolysaccharide (LPS)-stimulated RAW 264.7. LPS+: positive control—cells stimulated with LPS and treated with vehicle (DMEM with 2% of ethanol). LPS−: negative control—cells not stimulated with LPS and treated with vehicle. HEMNL: Hydroethanolic Extract of *Morus nigra* leaves. Data represent mean ± standard deviation. Statistical analysis was performed by one-way ANOVA test followed by Tukey’s post-test. * *p* < 0.023 in relation to LPS+; # *p* = 0.047 in relation to HEMNL 300 + GW 6471.

The LPS stimulation was also effective in increasing TNF-α production. Treatment with the extract significantly reduced (*p* < 0.0231) this cytokine by 25 to 68% at concentrations of 50 to 400 μg/mL. After concomitant treatment with the extract and the PPAR-α antagonist, GW 6471, a reduction in TNF-α was observed, but it was lower (*p* = 0.0469) than that caused by extract alone treatment. Meanwhile, the concomitant treatment with the extract and PPAR-γ antagonist, GW 9662, showed no significant difference in relation to treatment with the extract only (*p* > 0.999) (Figure 8).

## 3. Discussion

*Morus nigra* leaves are mainly constituted of phenolic compounds [26,27,28,29,30,31], primarily known for their anti-oxidant capacity, which is essential to help fight various diseases [32,33]. Since reactive oxygen species (ROS) can produce more inflammatory mediators and cause cellular damage, this ability of phenolic compounds is relevant in the treatment of inflammatory diseases. Furthermore, studies suggest that they are potent inhibitors of the COX enzyme, responsible for catalyzing the reaction of prostaglandin production in the inflammatory response [33].

The compounds rutin and isoquercitrin, identified in the HEMNL, are frequently reported in the leaves of this species [21,22,26,27,28,29,30]. In contrast to other studies, which suggested chlorogenic acid as the major compound [26,28,30,31], this compound was not found in HEMNL. These variations in the composition may be associated with the stage of plant development and seasonal variations. In addition, the plant material’s drying process, reparation, and extraction solvent can generate different phytochemical profiles, since the compounds extracted depend on the technique used and their affinity with the solvent [32].

Concerning safety, the HEMNL did not show cytotoxicity at the concentrations used in the anti-inflammatory tests. The present results follow a previous work showing the extract of *M. nigra* leaves presented low cytotoxicity in murine melanoma (B16F10), human keratinocyte (HaCat), murine fibroblast (L-929), and liver cancer (HepG2) strains [20,34]. In addition, the extract from *M. nigra* fruits has also been tested in the RAW 264.7 cells and showed low toxicity [35].

PPAR are nuclear receptors that play key roles in metabolism and cell differentiation. While PPAR-α acts mainly on fatty acid oxidation, promoting lipid-lowering effects, PPAR-γ is more associated with lipid storage and insulin sensitization [36,37,38]. Besides that, recently, the nuclear receptors PPAR have been considered a pharmacological target for developing anti-inflammatory drugs due to their ability to inhibit the expression of inflammatory cytokines and promote the differentiation of immune cells [9,10].

Considering the role of these receptors in lipid and glucose metabolism and their anti-inflammatory effects, dual PPAR-α/γ agonists may be beneficial in chronic diseases exacerbated by inflammatory processes. Thus, these drugs appear to maximize beneficial effects and reduce adverse effects caused by PPAR-γ activation [11,39].

HEMNL showed agonist activity for both PPAR subtypes (α/γ), even superior to the respective full agonists (WY 14642 and rosiglitazone, respectively). Isoprenylated flavonoids from the hydroethanolic extract of *M. nigra* twigs were tested for activation of PPAR-γ, and only sanggenol F, nigrasin K, and two unidentified compounds, among the ten compounds tested, showed PPAR-γ agonist activity. However, it was lower than that induced by rosiglitazone [40].

As the PPAR functioning depends on the formation of the heterodimer with the RXR receptor [38,41], transcriptional activity inhibition was also investigated when performing the simultaneous treatment with the extract and the specific antagonist. Therefore, it was possible to rule out the possibility that the activation is caused only by the interaction of HEMNL and the RXR receptor.

The extract was still able to activate PPAR-γ after concomitant treatment with the specific antagonist (GW 9662), probably resulting from its interaction with the RXR receptor. However, to achieve the maximum effect, comparable to rosiglitazone, the extract still depends on its binding to PPAR-γ. Concerning PPAR-α, the HEMNL effect seems to rely exclusively on its interaction with the PPAR-α, and not with the RXR.

Regarding the role of major compounds in the activation of receptors, the higher activation rate of HEMNL, in relation to the isolated substances, may be due to another compound not identified or the result of the synergistic effect between its compounds, demonstrating, in this case, the advantage of using the crude extract. Although the trend of research on medicinal plants has mainly focused on the purification and isolation of new compounds, the study of crude extracts is still of great public health relevance [42]. Its use is even recommended by WHO, mainly in the prevention and management of chronic diseases associated with lifestyle, which includes illnesses associated with a general inflammatory state [12,13].

In contrast, molecular docking studies suggested that rutin have favorable interaction with PPAR α and γ, showing even higher binding energy than thiazolidinediones [43,44]. In relation to isoquercitrin, no studies were found on its relationship with PPAR.

The anti-inflammatory capacity of *M. nigra* leaf extract was verified by assays conducted in murine macrophage cells (RAW 264.7) stimulated with LPS, an endotoxin present in the cell wall of Gram-negative cells, which is a strong trigger of the inflammatory response. Its recognition by Toll Like Receptors (TLR) triggers a signaling pathway that results in the activation of AP-1 and NF-kB transcription factors [45], which induces increased production of pro-inflammatory cytokines such as TNF-α and the enzyme inducible nitric oxide synthase (iNOS) [46,47,48,49]. Despite being most frequently associated with acute inflammation, LPS also plays an important role in maintaining chronic inflammatory diseases [50].

Another effect resulting from the inflammatory process triggered by LPS is the activation of the enzyme NADPH oxidase, which is responsible for the increase in the production of ROS [51,52]. The intracellular ROS reduction in LPS-stimulated cells, caused by the treatment with HEMNL, confirms the anti-oxidant potential of *M. nigra* leaves, being the first study to evaluate this activity using the DCF method in this model. The anti-oxidant activity of *M. nigra* leaves has already been evidenced in other studies by different methods and different situations, such as in vitro, by the uptake of the DPPH and ABTS radical; in vivo by the evaluation of the activity of catalase and superoxide dismutase enzymes; by reactive substances to thiobarbituric acid; and by the recovery of reduced glutathione levels [30,31,53,54,55].

An anti-oxidant agent can delay or prevent the oxidation of substrates, minimizing damage to tissues and organs caused in various pathological processes. As inflammation is related to oxidative stress, since one can mutually induce the other, an extract with anti-oxidant capacity may be useful in treating inflammatory diseases [56,57,58].

The anti-oxidant activity may be related to the presence of rutin and isoquercitrin, compounds belonging to the flavonoid group, recognized for its high and well-established anti-oxidant capacity [32,33].

A low amount of nitric oxide, produced by the enzyme iNOS, is important for eliminating pathogens during the inflammatory response. However, the exacerbated production can contribute to developing several pathologies [59,60].

For example, the systemic release of NO inhibits the migration of neutrophils to the site of infection, making it difficult to remove bacteria and facilitate sepsis. In arthritis, NO contributes to joint damage and bone mass degradation [60]. In addition, high levels of NO can cause DNA damage, cell proliferation, and angiogenesis and are often associated with tumors with poor prognosis [61]. Thus, decreasing NO is a pharmacologically interesting effect of new anti-inflammatory drugs.

TNF-α is one of the main cytokines mediating the acute and chronic inflammatory response. At the site of inflammation, it stimulates the production of chemokines and other mediators and activates endothelial cells to express adhesion molecules. Thus, more leukocytes are recruited to the site of inflammation. In addition to acting locally, TNF-α can enter the bloodstream and cause systemic effects such as stimulating the production of acute phase proteins by the liver, the release of adrenocorticotropic hormone, falling blood pressure, fever, myalgias, and others [62,63].

Under the conditions presented in this study, HEMNL reduced the production of NO and TNF-α by more than 50%, revealing its potential as an anti-inflammatory agent. Furthermore, this effect was significantly reduced in the presence of the specific PPAR-α antagonist, suggesting the need to activate this receptor in the mechanism of action of the hydroethanolic extract of *M. nigra*. The same was not observed in concomitant treatment with the specific PPAR-γ antagonist.

In comparison, another study that evaluated the nuciferine, a natural alkaloid, which also showed agonist activity towards PPAR α/γ, reduced the production of TNF-α in LPS-stimulated RAW 264.7 cells by 32% [64].

The aqueous extract of *M. nigra* leaves was also evaluated for production of NO inhibition in the serum or brain of mice treated for 1 or 7 days at doses of 10, 30, and 100 mg/kg. Despite not showing differences in the acute treatment (1 day), after the subchronic treatment (7 days), the extract showed a reduction of NO from 29 to 41% in the serum and 15 to 27% in the brain [54]. Regarding the decrease in TNF-α, a study carried out in mice with sepsis induced by LPS also observed a higher survival rate and a significant reduction in the production of TNF-α in the group treated with the *M. nigra* leaves extract [65].

The action of PPAR resulting from activation by HEMNL may be one of the mechanisms responsible for the anti-inflammatory effects observed, since they can act inhibiting the pro-inflammatory transcription factors NF-kB e AP-1, resulting in decreased cytokines and oxidative stress [9,10].

Besides that, other studies show the participation of rutin and isoquercitrin, through other mechanisms, in the anti-inflammatory process. In a study conducted with mice, rutin was able to reduce the inflammatory mediators IL-1, TNF-α, and IL-18, probably by inhibiting the signaling pathway of Toll-like receptors and the P2X7 receptor [66].

In activated neutrophil cells, rutin significantly reduced the production of TNF-α, nitric oxide and the enzyme myeloperoxidase [67]. While in rats with nephrotoxicity caused by the drug carfilzomib, rutin reduced oxidative stress and inflammation possibly by inhibiting the NF-KB signaling pathway [68]. Its oxidative stress-reducing and anti-inflammatory effects have also been visualized in rats with fluoride-induced neurotoxicity [69] and in rats with methanol-induced optic neuropathy [70].

Isoquercitrin showed a hepatoprotective effect in mice with acetaminophen-induced hepatotoxicity. Part of this effect was attributed to the reduction in the production of iNOS, TNF-α, IL-1β, and IL-6, through the NF-KB pathway [71]. The same effects were observed in mice with LPS-induced cardiac dysfunction [72]. Considering these studies and several others that elucidate the anti-inflammatory capacity of rutin and isoquercitrin, the effects of the extract could be attributed, at least in part, to the presence of these compounds.

## 4. Materials and Methods

### 4.1. Reagents and Standards

Cell lines HeLa and RAW 264.7 were purchased from the Rio de Janeiro Cell Bank (BCRJ). Rutin, isoquercitrin, chlorogenic acid, rosiglitazone, WY 14642 {[4-chloro-6-(2,3-xylidino)-2-pyrimidinyl- thio]acetic acid}, GW 9662 (2-chloro-5-nitro-N-phenylbenzamide), GW6471 {N-[(2S)-3-[4-[2-(5-methyl-2-phenyl-1,3-oxazol-4-yl)ethoxy]phenyl]-2-[[(Z)-4-oxo-4-[4-(trifluoromethyl)phenyl]but-2-en-2-yl]amino]propyl]propanamide}, MTT [3-(4,5-dimethylthiazol-2-yl)-2,5-diphenyl tetrazolium bromide], lipopolysaccharide from Escherichia coli, 2′,7′-dichlorofluorescin diacetate, Griess reagent, and mouse Tumor Necrosis Factor α Elisa kit were purchased from Sigma-Aldrich (St. Louis, MO, USA). Lipofectamine was purchased from Thermo Fisher, Invitrogen (Waltham, MA, USA). Dulbecco’s modified Eagle’s medium (DMEM) and Fetal Bovine Serum (FBS) were purchased from Gibco—Thermo Fisher Scientific. For HPLC analysis, acetonitrile, phosphoric acid, and methanol were purchased from Tedia (Fairfield, OH, USA).

### 4.2. Plant Material and Extraction Procedure

The leaves of *Morus nigra* L. were collected in October 2017 (fruiting period), in the afternoon, in Brasilia-DF (Brazil). The botanical material was identified by the botanist Assoc Prof Christopher W. Fagg from the Institute of Biology of the University of Brasília, according to the voucher specimen (Fagg CW 2302) deposited in the Herbarium of the University of Brasília.

The access to Genetic Heritage was approved by CGEN (Genetic Heritage Management Council), registered at SISGEN (National System for the Management of Genetic Heritage and Associated Traditional Knowledge) under the number A215A9A.

The hydroethanolic extract of *Morus nigra* leaves (HEMNL) was prepared by passive cold maceration according to previous extract standardization described by [20]. The mass of the pulverized leaves used in the extractive process was 1468.22 g. At the end of the process, after lyophilization, a mass of 155.58 g was obtained. Thus, the yield was 10.59%.

### 4.3. HPLC-DAD Analysis

HPLC-DAD analysis was conducted using the chromatograph LaChrom Elite (Hitachi, Tokyo, Japan) equipped with an L2455 DAD detector adjusted in the range of 230 to 400 nm, L2200 injector, L2130 pump, L2300 column oven, and a C18 column (150 × 4.6 mm, 5 µm, Merck, Darmstadt, Germany). Data were obtained with EZChrom Elite software, version 3.3.2 SP1 (Santa Clara, CA, USA). Analysis conditions were according to [20]. The mobile phase consisted of a gradient of 1% phosphoric acid and acetonitrile (Table 1) with a flow rate of 0.5 mL per minute.

Samples were obtained by diluting the extract with methanol to the concentration of 4 mg/mL followed by filtration with a 0.45 μm filter (Millex, Merck KGaA, Darmstadt, Germany). Commercial standards were used in an attempt to identify the compounds present in HEMNL by comparison of retention times and ultraviolet spectra. The standards were caffeic acid, chlorogenic acid, ferulic acid, kaempferol, gallic acid, rosmarinic acid, ellagic acid, isoquercitrin, hesperetin, quercetin, resveratrol, vitexin, isovitexin, coumarin, and myricetin acquired from Sigma-Aldrich^®^, hyperoside acquired from Hwi Analytik Gmbh^®^ (Rülzheim, Germany), and rutin from Chromadex^®^ (Los Angeles, CA, USA).

### 4.4. Cell Culture

Cell lines HeLa and RAW 264.7 were grown in high glucose Dulbecco’s modified Eagle’s Medium (DMEM) supplemented with 10% fetal bovine serum, 1 mM sodium pyruvate, and 1% antibiotics (penicillin-streptomycin). Cells were incubated at 37 °C and 5% of CO_2_ in a humidified atmosphere. The assays were carried out between the third and tenth passages.

### 4.5. Cell Viability Assay

Cells were seeded in a 96-well plate at the density of 0.8 × 10^4^ cells/well for HeLa and 1 × 10^5^ cells/well for RAW 264.7. After 24 h of incubation, the cells were treated with MNEH at concentrations ranging from 1000 µg/mL to 50 µg/mL. Additionally, RAW 264.7 cells were tested with or without LPS (1 µg/mL) stimulation 3 h after the treatment. The negative control consisted of treatment with 2% ethanol (solvent used for extract dilution).

Following 24 h after treatment, cell viability was assessed by MTT assay described by Mosmann (1983) with modifications [73]. Cells were incubated with MTT (1 mg/mL) for 4 h and then solubilized with acidified isopropanol. The absorbance was measured at 570 nm, and the cell viability was calculated using the following equation:

(1) $$\mathrm{Cell}\;\mathrm{viability}\;(\%)=\frac{\mathrm{Absorbance}\;\mathrm{of}\;\mathrm{experimental}\;\mathrm{group}}{\mathrm{Absorbance}\;\mathrm{of}\;\mathrm{negative}\;\mathrm{control}}\times 100.$$

### 4.6. PPAR Luciferase Reporter Assay

HeLa cells were seeded in a 48-well plate at a concentration of 4 × 10^4^ cells/well in DMEM medium with 10% FBS and without antibiotics. The cells were incubated for 24 h and then submitted to transfection with the Lipofectamine 2000^®^ (Waltham, MA, USA) (Transfection reagent) according to the manufacturer’s protocol. The transfection occurred with a solution of liposomes formed with 60 ng of a plasmid containing the nuclear receptor (PPAR-γ or PPAR-α), 240 ng of the LUC responsive element, and 0.5 µL of Lipofectamine 2000^®^ in DMEM without FBS and antibiotics. The plasmids used were (i) cDNA for the human PPARγ receptor (pCMV-SPORT6-PPARγ); its responsive element was fused to the luciferase reporter gene; (ii) PPARα LBD fused to the Gal4 DNA-binding domain; (iii) a plasmid containing the luciferase reporter gene under regulation by five Gal4 DNA-binding elements. These plasmids were kindly provided by Dr. Paul Webb from the Methodist Research Institute, TX, United States. After 6 h of incubation at 5% CO_2_ at 37 °C, the contents of the well were removed and replaced by the treatment with HEMNL, rutin, isoquercitrin, or the positive controls, rosiglitazone (PPAR-γ agonist) or WY 14642 (PPAR-α agonist). Additionally, the concomitant treatment with HEMNL and the specific antagonists, GW 9662 (PPAR-γ antagonist) or GW 6471 (PPAR-α antagonist), was tested.

### 4.7. Measurement of Intracellular ROS

The RAW 264.7 cells were seeded in a 96-well plate at 1 × 10^5^ cells/well density. After 24 h, cells were treated with HEMNL at a concentration of 50, 100, 200, 300, or 400 µg/mL and stimulated with LPS (1 µg/mL) 3 h later. Following 24 h after the treatment, cells were washed with PBS and incubated for 30 min at 37 °C and 5% CO_2_ with DCFH-DA 40 µM. Finally, cells were washed, and PBS was added. The fluorescence was measured in a microplate reader set at 485 nm excitation and 535 nm emission. The results were normalized by mg of protein and quantified by a BCA kit as per the manufacturer’s instructions.

### 4.8. Nitrite Determination

The nitrite concentration was determined by the Griess method. RAW 264.7 cells were seeded in 96-well plate at a density of 1 × 10^5^ cells/well. Following 24 h after incubation, cells were treated with HEMNL at a concentration of 50, 75, 100, 150, or 200 µg/mL (higher concentrations were excluded due to color interference in the analysis) and stimulated with LPS (1 µg/mL) 3 h later. After 48 h, 100 µL of cells supernatant was mixed with the same volume of Griess reagent. The absorbance was measured, after 10 min, at a wavelength of 540 nm. Nitric oxide concentration was calculated using a sodium nitrite standard curve.

### 4.9. TNF-α Determination

RAW 264.7 cells were seeded in 96-well plate at a density of 1 × 10^5^ cells/well. Following 24 h after incubation, cells were treated with HEMNL at a concentration of 50, 100, 200, 300, or 400 µg/mL and stimulated with LPS (1 µg/mL) 3 h later. The TNF-α was determined with a Mouse TNF-α ELISA kit, according to the manufacturer’s instructions.

### 4.10. Statistical Analysis

All results were represented by the average of three independent experiments performed in triplicate. Statistical analyzes were performed using the GraphPad Prism 6^®^ program. The results obtained were compared by one-way analysis of variance (one-way ANOVA) followed by the Tukey test for multiple comparisons or by the Mann–Whitney test for comparing two groups. A significance level of *p* < 0.05 was adopted.

## 5. Conclusions

This is the first study to demonstrate the extract of *M. nigra* leaves as an agonist of the two types of PPAR, α and γ, probably due to the synergistic effect of its compounds, since its major compounds, rutin and isoquercitrin, were not able to significantly activate the receptors. In addition, the extract showed good anti-inflammatory activity, significantly reducing the production of ROS, NO, and TNF-α, in LPS-stimulated RAW 264.7 cells.

The specific antagonist of PPAR-α, the compound GW 6471, was able to partially block the effect of the extract, demonstrating the need for activation of the PPAR-α to obtain the anti-inflammatory effects of HEMNL. However, further studies are needed to confirm the mechanism of the anti-inflammatory action of *M. nigra*.

## Figures and Tables

**Table 1:** HPLC-DAD analysis elution gradient.

Time (min)	Phosphoric Acid 1% (%)	Acetonitrile (%)
0	90	10
40	70	30
45	90	10
